# Late antenatal care booking and associated factors among pregnant women in Mizan-Aman town, South West Ethiopia, 2021

**DOI:** 10.1371/journal.pgph.0000311

**Published:** 2023-01-05

**Authors:** Gelila Gashawbeza Battu, Roza Teshome Kassa, Haweni Adugna Negeri, Leul Deribe kitawu, Kassahun Demelash Alemu

**Affiliations:** 1 Mizan Aman Health Science College, Aman, South West Ethiopia; 2 School of Nursing and Midwifery, College of Health Science, Addis Ababa University, Addis Ababa, Ethiopia; PLOS: Public Library of Science, UNITED STATES

## Abstract

**Background:**

Antenatal care (ANC) is one of the most important ways to reduce maternal and neonatal morbidity and mortality. According to data from poor countries, the majority of pregnant women attend ANC when they are in their later stages of pregnancy. In this regard, limited information is currently known about the factors that determine ANC scheduling and the type of care for pregnant women in the town of Mizan-Aman in southwestern Ethiopia. Therefore, the purpose of this study was to determine late antenatal care booking and associated factors among pregnant women in the Town.

**Method:**

The institutional-based cross-sectional study design was conducted in Mizan-Aman town using a systematic random sampling method through structured questions from February 15 to March 25, 2021. The collected data was entered into EPI info-7 which later on, was exported to SPSS version 20 for statistical analysis. Binary and multiple logistic regressions were used to identify associated factors and p-value <0.05 was considered for statistical significance.

**Results:**

A total of 425 female pregnant women participated, making a 100% response rate. The prevalence of delayed first ANC bookings in this study was 70.0% [95.0%, CI = 65.65–74.35]. Multivariate analysis revealed that unplanned pregnancy [AOR = 2.63, 95% CI: 1.18, 5.85], inappropriate perception of ANC starting time [AOR = 4.1, 95% CI: 1.9, 8.83], pregnant women who were unaware of pregnancy-related danger signs [AOR = 6.76, 95% CI: 2.83, 16.1], and pregnant women who were unaware of service delivery during working hours in the institution [AOR = 0.44, 95% CI: 0.19, 0.98].

**Conclusion:**

The current study showed a greater prevalence of delayed ANC beginnings, and the factors for this were having an unplanned pregnancy, lack of awareness about pregnancy danger signs, inappropriate perception of ANC starting time, and pregnant women who were unaware of service delivery during working hours at the institution. Responsible bodies working in maternal and child health care better create awareness of the benefits of early ANC booking and appropriate ANC starting times. Further, each health institution’s MCH clinic should deliver the service through working hours.

## Introduction

### Background

The provision of antenatal care (ANC) services is one of the most important interventions for improving maternal health. ANC services assist pregnant women and adolescent girls in ensuring the best health conditions for both mother and baby during pregnancy by utilizing skilled healthcare professionals [1]. One of the key strategies for reducing maternal and neonatal morbidity and mortality is through preventing, detecting, alleviating, or managing pregnancy-related health problems that may affect mothers and babies. These are complications of pregnancy that worsen preexisting conditions during pregnancy and effects of unhealthy lifestyles, but this can be achieved by early booking within the first trimester [2].

Globally, around 210 million women become pregnant every year. There are 31 million stillbirths, 80 million unwanted pregnancies, and 47,000 women who die as a result of unsafe abortions [3]. The global estimation of pregnant women who receive an ANC was 71%. In sub-Saharan Africa, 69% of pregnant women have at least one ANC visit. Whereas the coverage of at least four ANC visits was lower, which was 44%. Of this, around 80% of women in the richest quintile have access to three or more ANC visits, while only 48% of the poorest women have the same level of access [4]. Most pregnant women were late in scheduling their first ANC appointment due to a lack of knowledge about when to begin their first ANC visit, the number of recommended visits, and a lack of familiarity with pregnancy symptoms [5].

According to the 2016 World Health Organization (WHO) recommendations, a healthy pregnancy requires a minimum of eight ANC visits. The first contact, also known as early prenatal care, should be during the first trimester; two contacts, at 20 and 26 weeks of pregnancy, should be during the second trimester; and five contacts during the third trimester (at 30, 34, 36, 38, and 40 weeks) [6]. In our country, the first ANC booking is acceptable until the baby is 16 weeks old [7].

The provision of ANC services by trained health professionals at healthcare institutions has a substantial influence on lowering the risks of morbidity and mortality for mothers and children. A timely ANC booking could save both the mother’s and the children’s lives [8]. Furthermore, it includes nutrition and health checks, counseling, and support for women and their families, and a higher likelihood of delivery in the presence of skilled birth attendants, leading to fewer maternal and fetal deaths [4,9].

Globally, the rate of child mortality decreased from 90 to 43 deaths per 1,000 live births between 1990 and 2015; the same maternal mortality ratio declined by 44% per 100, 000 live births. The majority of these deaths are from preventable causes and treatable diseases [10]. Therefore, knowing the coverage and associated factors of ANC in different settings help to design effective strategy. Based on our search abilities in our study settings, there has never been a study conducted before regarding the associated factors and prevalence of late ANC booking. Therefore, this study is aimed at determining the prevalence of late ANC initiation and factors associated with it among pregnant women attending antenatal clinic in Mizan-Aman town.

## Methods

### Study design

An institutional-based cross-sectional study design was used to assess late antenatal care booking and associated factors among pregnant women in Mizan-Aman town, southwest Ethiopia, in 2021.

### Study area and period

The study was conducted in Mizan-Aman, a town in Ethiopia’s Bench-Sheko Zone in the southwest, which is located 574 km away from the capital city of Addis Ababa. There are five health posts, one health institution, and one teaching hospital in the town. The town is divided into five kebeles. Coffee is the mainstay of the town’s economy. The data was collected between February 15 and March 25, 2021.

### Source population

All mothers attending antenatal clinics at Mizan-Aman town health institutions.

### Study population

Pregnant mothers receive ANC services at the Mizan-Aman Town health facility during the data collection period.

### Sample size determination

The sample size for the first objective was calculated using a single population proportion formula assuming a 95% confidence interval (CI), 5% marginal error (d), and the expected proportion of late ANC booking was 59.4% [11], which yielded a sample size of 370. The sample size for the second objective was determined by using Epi-Info ^TM^ 7 software assuming 95% CI, power of 80%, two significantly associated variables from other studies i.e., maternal age (OR = 3.09) [12] and Perceived ANC is the starting time (OR = 3.4) [13] which yields a sample size of 376 and 386. Accordingly, the largest sample was obtained from the second objective and adding a 10% non-response rate, the final sample size for this particular study was 425.

### Sampling technique

**Fig 1:**
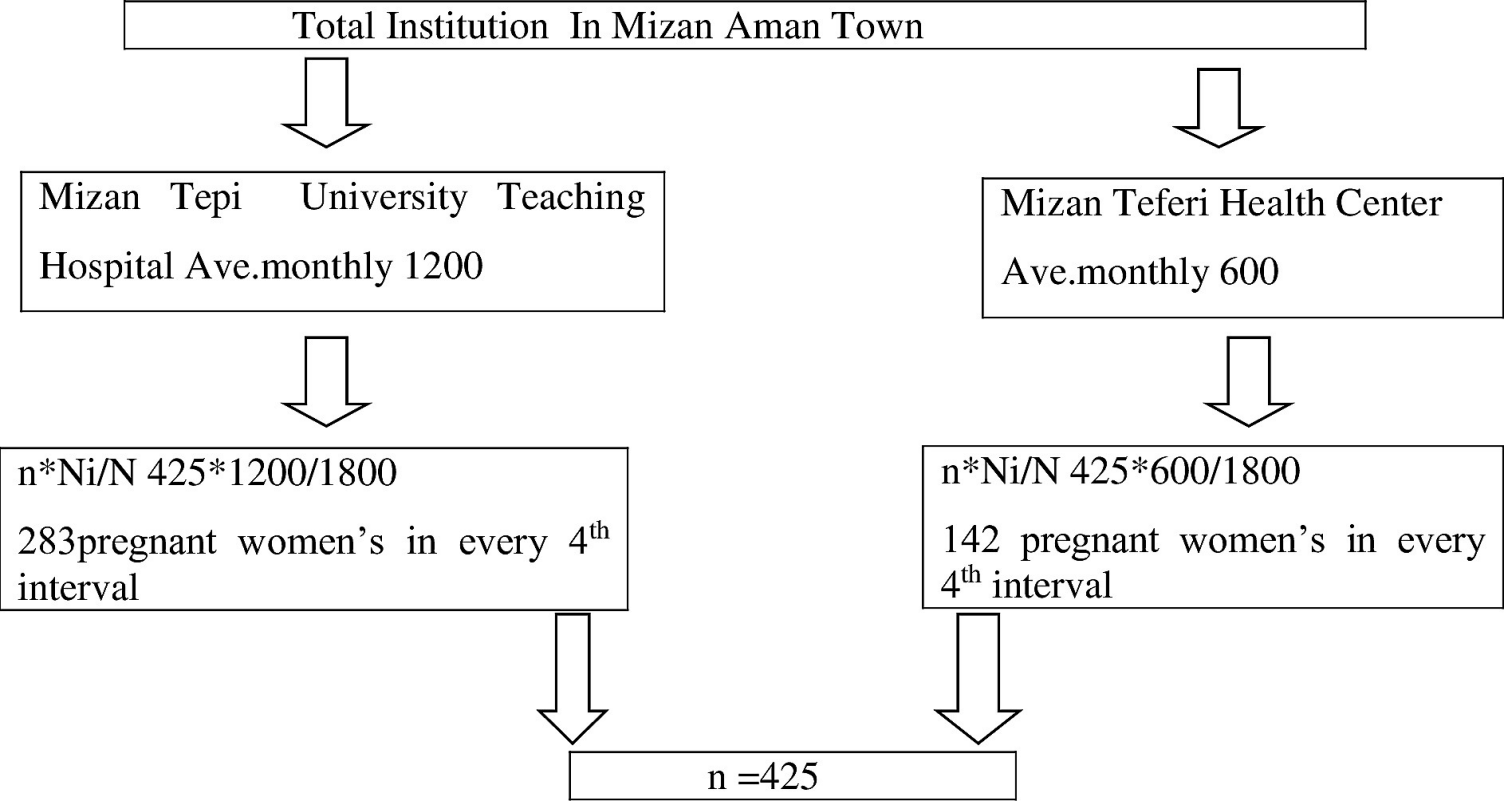
Schematic representation of sampling techniques on timing of first ANC booking and associated factors among pregnant women in Mizan-Aman town, southwest Ethiopia 2021.

The participants were recruited from public health facilities in Mizan-Aman town, southwest Ethiopia through a systematic random sampling technique. The town has two health facilities that are providing ANC services. The expected number of pregnant mothers who visited the health institutions during the study period was calculated based on the number of clients who visit the hospital and the health center for the previous three months (three-month report of each) i.e., 1,200 and 600 for Mizan Tepi University Teaching Hospital and Mizan Teferi Health center, respectively. A calculated sample size of 425 was allocated proportionally for each; for Mizan tepi university teaching hospital 283 and from Mizan teferi health center 142 pregnant women’s was interviewed in the 4th interval (Fig 1).

### Study variables

Late initiation for ANC visit was the outcome variable whereas socio-demographic variable (residence, marital status, education, occupation and income, husband education, occupation), obstetric-related factors (parity, means of pregnancy recognition, type of pregnancy, previous history of complication), pregnant women related factors (advice from others, Knowledge about ANC) and health care facility related factors (transport cost, health care provider behavior, communication advice, distance) where the independent variables.

### Operational definition

**Early booking of antenatal care**: When pregnant women arrived at a healthcare facility before 16 weeks of gestation, they were considered to have booked an ANC appointment late.

**Late booking of antenatal care**: When pregnant women arrived at a healthcare facility at or after 16 weeks of gestation, they were considered to have booked an ANC appointment late [7].

**Perceived timing of ANC**: mothers who thought the first ANC booking should start before 4 months of gestation were thought to have an appropriate perception of ANC booking, whereas mothers who thought the first booking should start after 4 months of gestation were thought to have an inappropriate perception of ANC booking [13].

**Transportation system**: accessibility of public transportation to the healthcare facility.

### Data collection tools and procedure

The data collection was conducted through face-to-face interviews using a structured questionnaire adapted from different literature [13–15], which consisted of six-parts such as: demographic information, obstetric history, history of the current pregnancy, the benefit of the ANC, knowing danger signs, and knowledge of ANC utilization. Illness experience and perceived susceptibility to pregnancy-related health problems: health-care-related factors influencing late ANC initiation. A structured survey questionnaire was written in English and translated into Amharic. The Amharic tool was retranslated in reverse to the original one by language experts to check for its reliability. Full informed consent was obtained from all eligible participants explaining the objectives of the study to participants in their language. After they obtained informed consent, the interview was conducted in a private room after they received the service in the health facility. All respondents’ medical charts were reviewed on the same day to collect additional information on gestational age after having consented. Three interviewers (BSc midwives) speaking Bench and Amharic and two supervisors were recruited from another health facility to fill out the questionnaire under the supervision of employed (BSc HO and BSc midwifery supervisors). The supervisors provide all items necessary for the data collection day, check the questionnaire for completeness and consistency, and solve the problem during data collection.

### Data quality assurance

Based on the feedback from the pre-tested questionnaire, we made adjustments to the appropriateness of the data collection instrument. Data collectors also received one-day training on how to conduct the questionnaire and check for accuracy. To guarantee that the data is complete, the lead investigator and supervisors have been closely monitoring the data collected daily. Data coding and data entry were double-checked throughout the process.

### Data analysis

To enter the data, an Epidata manager version of 4.4.2.1 was used, which was then exported to the statistical Package for Social Sciences (SPSS) version 20.0 software package for statistical analysis. Descriptive statistics of frequencies and percentages were generated for categorical variables and supplied in the form of figures, tables, and texts. The bivariate and multivariate binary logistic regression models were used to determine the connection between the factors and late ANC booking. In bivariate and multivariate logistic regression, a variable with a P-value of 0.05 was considered a strongly associated outcome variable.

#### Ethical consideration

Ethical clearance was obtained from Addis Ababa University College of Health Science Institution Review Board (IRB). Support letter was obtained from the Health Bureau of Bench Shako Zone and respective health institutions before field activity started. Written informed consent was obtained from the study participants after each of the study participants (pregnant women) were informed about the purpose, methods of collection, anticipated benefit, and risk of the study by the data collectors. Privacy and confidentiality were maintained throughout data collection, analysis, and result dissemination.

For COVID-19 prevention and control, all data collectors and supervisors were provided face covers, hand washing soap and water and hand sanitizer gel, while the pregnant women already use face covers when visiting antenatal consultations.

## Result

In this study, 425 pregnant women were enrolled with more than 263(61.9%) live in urban. The majority of participants, 302 (71.1%), were between the ages of 20 and 35, with pregnant women’s mean ages being 25.7 (+5.5 SD). About 381 (90%) of the moms were married, and 120 (28.2%) of the women were housewives. Regarding the mother’s educational background, the majority, 114 (26.8%), attended elementary school, and 89 (20.9%), had a college or higher education (Table 1).

**Table 1:** Socio-demography characteristics of participants who visit health institutions for ANC service in Mizan-Aman town, southwest Ethiopia 2021.

Variables	Frequency	Percent
**Residence**		
Urban	263	61.9
Rural	162	38.1
**Participant Age**		
<20	102	24.0
20–35	302	71.1
>35	21	4.9
**Maternal educational status**		
No formal education	110	25.9
Primary (1–8)	114	26.8
Secondary (9–12)	112	26.4
College and above	89	20.9
**Maternal occupational status**		
Government employee	98	23.1
Privet employee	54	12.7
Housewife	120	28.2
Students	71	16.7
Other (farmer)	82	19.3
**Marital status**		
Married	381	89.6
Single	24	5.6
Divorced	9	2.1
Widowed	11	2.6
**Husband educational status**		
No formal education	60	15.7
Primary (1–8)	66	17.3
Secondary (9–12)	111	29.1
College and above	144	37.8
**Husband occupational status**		
Government employee	142	37.3
Privet employee	31	8.1
Private business	86	22.6
Daily laborer	34	8.9
Other (farmer)	88	23.1
**Monthly estimated income**		
<400	8	2.0
400–1000	58	14.4
>1000	337	83.6

### Obstetrics history of the study participants

Among the total participants, 270 (63.5%) were multigravida and about 155 (36.5%) were primigravida. Stillbirths and child deaths accounted for around 49 (20%) of the total multigravida abortions, of which 198 (73.3%) and 144 (58.8%) had a history of cesarean delivery (Table 2).

**Table 2:** Obstetrics history of the study participant’s visits to the health institution for ANC service in Mizan-Aman town, southwest Ethiopia 2021.

Variables	Time of ANC initiation		Total
	Late n (%)	Early n (%)	
**Gravida**			
Primi-gravida	105(35.4)	50(39.1)	155(36.5)
Multi-gravida	192 (64.6)	78 (60.9)	270 (63.5)
**Children alive**			
Yes	167 (97.1)	71(97.3)	238(97.1)
No	2 (2.7)	4 (2.9)	7 (2.9)
**Children died**			
Yes	39 (22.7)	10 (13.7)	49 (20.0)
No	133 (77.3)	63 (86.3)	196 (80.0)
**Stillbirth**			
Yes	38 (22.1)	11 (15.1)	49 (20.0)
No	134 (77.9)	62 (84.9)	196 (80.0)
**History of abortion**			
Yes	57 (29.7)	15 (19.2)	72 (26.7)
No	135 (70.3)	63 (80.8)	198 (73.3)
**A Problem with the last delivery**			
Yes 52(31.5)		23(30.2)	75(30.6)
No 120(68.5)		50(69.8)	170(69.4)
**History of cesarean delivery**			
Yes 71(41.5)		30(40.5)	101(41.2)
No 100(58.5)		44(59.5)	144(58.8)

### Respondent’s current pregnancy history

Among the study participants, 106 (24.9%) of pregnant women recognized their pregnancy by a urine test. The others found out that they were pregnant by a pregnancy sign, 90 (21.2%), and missed their menstrual period by 3 months, 85 (20.0%). Out of the total participants, 43% of women had booked their first ANC visit; the rests were two ANC visits and three ANC visits, with 34.8% and 13.9%, respectively.

A majority of the respondents’ pregnancies were planned, 282 (66.4%), and around 278 (98.6%) of them involved their husbands in the planning. Among the total respondents, 238 (56%) of pregnant women had received advice to start ANC service, and a majority of them had gotten advice from community health workers 99 (41.4%), and friends 53 (22.2%). Half of the pregnant women booked time for the ANC follow-up because they thought it was an appropriate time 206 (48.5%) (Table 3).

**Table 3:** History of current pregnancy and ANC of the study participants visiting health institution for ANC service in Mizan-Aman town, southwest Ethiopia 2021.

Variable	Timing of ANC initiation		Total
	Late n%	Early n%	
**Pregnancy knowing**			
Missed period once	13(4.4)	14(10.9)	27(6.4)
Missed period twice	57(19.2)	17(13.3)	74(17.4)
Missed period three and more	59(19.9)	26(20.3)	85(20.0)
Physiological changes	32(10.8)	8(6.3)	40(9.4)
Other signs like nausea	59(19.9)	31(24.2)	90(21.2)
By examination [urine test]	74(24.9)	32(25.0)	106(24.9)
Other	3(1.0)	0(0.0)	3(0.7)
**Receive antenatal care in this pregnancy**			
It is my first time	147(49.5)	35(27.3)	182(42.8)
Two times	94(31.6)	54(42.2)	148(34.8)
Three times	36(12.1)	23(18.0)	59(13.9)
Four times	11(3.7)	12(9.4)	23(5.4)
Greater than four	9(3.0)	4(3.1)	13(3.1)
**Planned pregnancy**			
Yes	174(58.6)	108(84.4)	282(66.4)
No	123(41.4)	20(15.6)	143(33.6)
**Planned include husband**			
Yes	171(98.3)	107(99.1)	278(98.6)
No	3(1.7)	1(0.9)	4(1.4)
**Wanted after conception**			
Yes	83(67.5)	20(100.0)	103(72.0)
No	40(32.5)	0(0.0)	40(28.0)
**Wanted by husband(partner) after conception**			
Yes	72(62.1)	17(77.3)	89(64.5)
No	44(37.9)	5(22.7)	49(35.5)
**Current pregnancy problem**			
Yes	86(29.0)	30(23.4)	116(27.3)
No	211(71.0)	98(76.6)	309(72.7)
**Receive advice to come to ANC in the current pregnancy**			
Yes	179(60.3)	59(46.1)	238(56.0)
No	118(39.7)	69(53.9)	187(44.0)
**From whom you get advice**			
Community health workers	72(40.2)	27(45.0)	99(41.4)
Husband	22(12.3)	9(15.0)	31(13.0)
Mother	21(11.7)	10(16.7)	31(13.0)
Sister	13(7.3)	5(8.3)	18(7.5)
Friend	47(26.3)	6(10.0)	53(22.2)
Other	4(2.2)	3(5.0)	7(2.9)
**Reason to decide to start follow- up at this time**			
Thought it was the appropriate time	112(37.7)	94(73.4)	206(48.5)
Found money	32(10.8)	7(5.5)	39(9.2)
Booking at convenience	97(32.7)	15(11.7)	112(26.4)
Given appointment for today	22(7.4)	2(1.6)	24(5.6)
Others	34(11.4)	10(7.8)	44(10.4)

### Timing of ANC attendance and reasons for late ANC booking service

**Fig 2:**
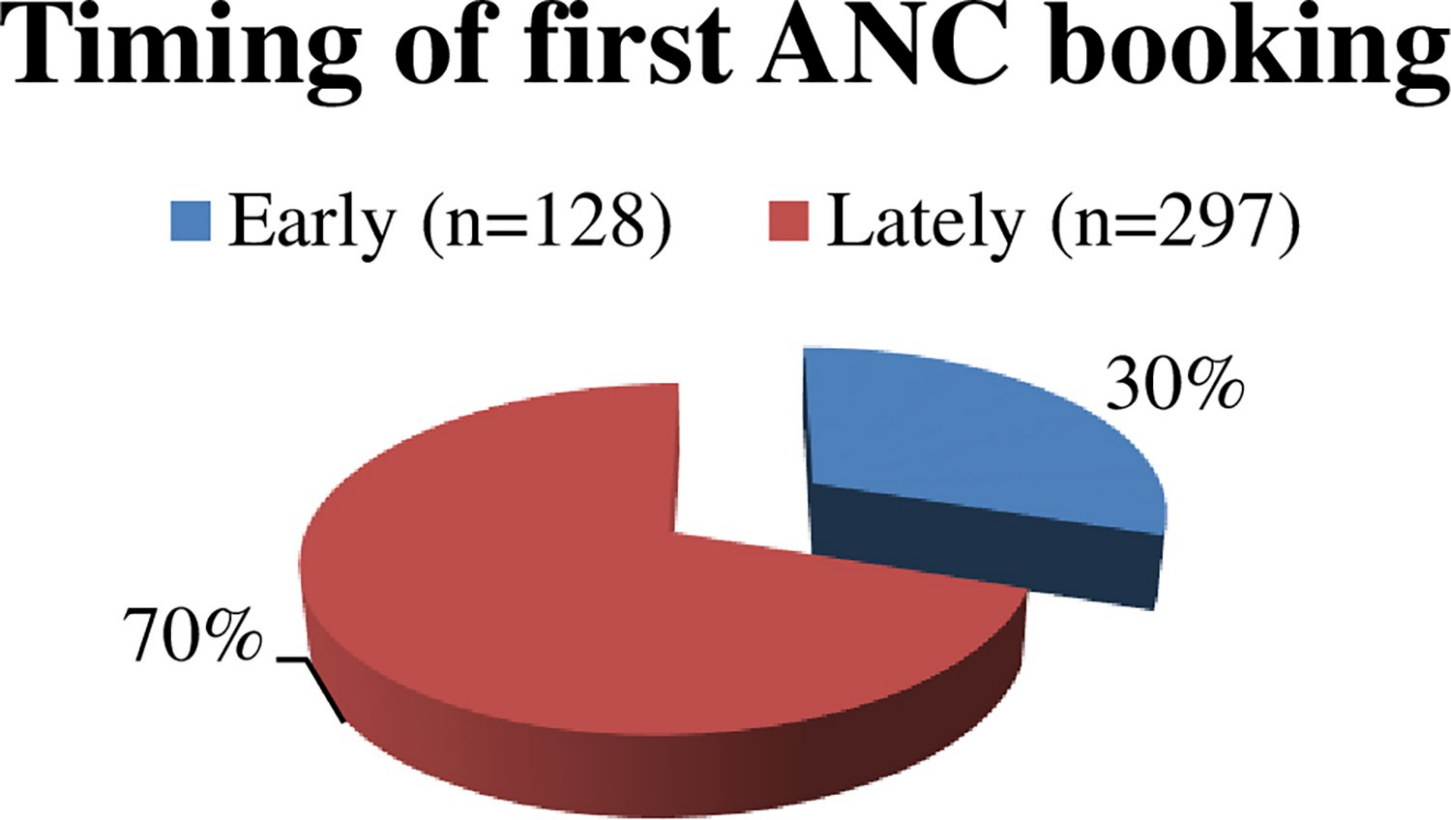
Timing of first Antenatal care booking among pregnant women in Mizan-Aman town, southwest, Ethiopia, 2021.

The majority of the pregnant women booked antenatal care services lately 297 (70%) whereas some 128 (30%) booked earlier (Fig 2).

**Fig 3:**
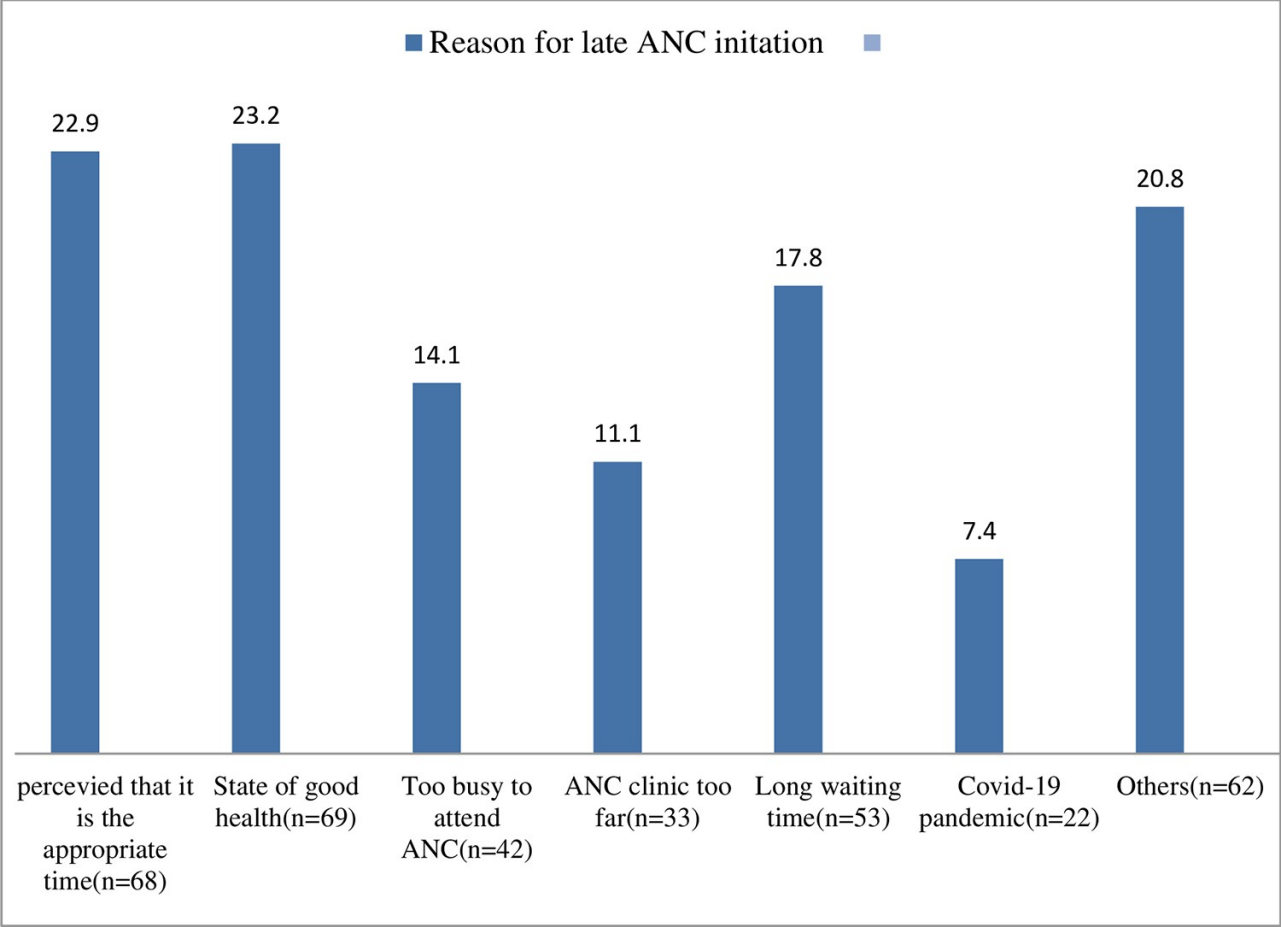
Reasons for late ANC attendance mentioned by the pregnant women in Mizan-Aman town, southwest, Ethiopia, 2021.

Expectant mothers were late for a variety of reasons. perceiving to be in the right place at the right time (22.9%), in good health (23.2%), having spent a lengthy period waiting in a health facility (17.8%), being too busy to visit ANC (14.1%), ANC clinic too far away (11.1%), perception of institutional closure due to the COVID-19 epidemic (7.4%), among others, unplanned pregnancy (7.1%), and appointment beyond 4 months (4%) (Fig 3).

### Participant knowledge about the time of ANC utilization

**Fig 4:**
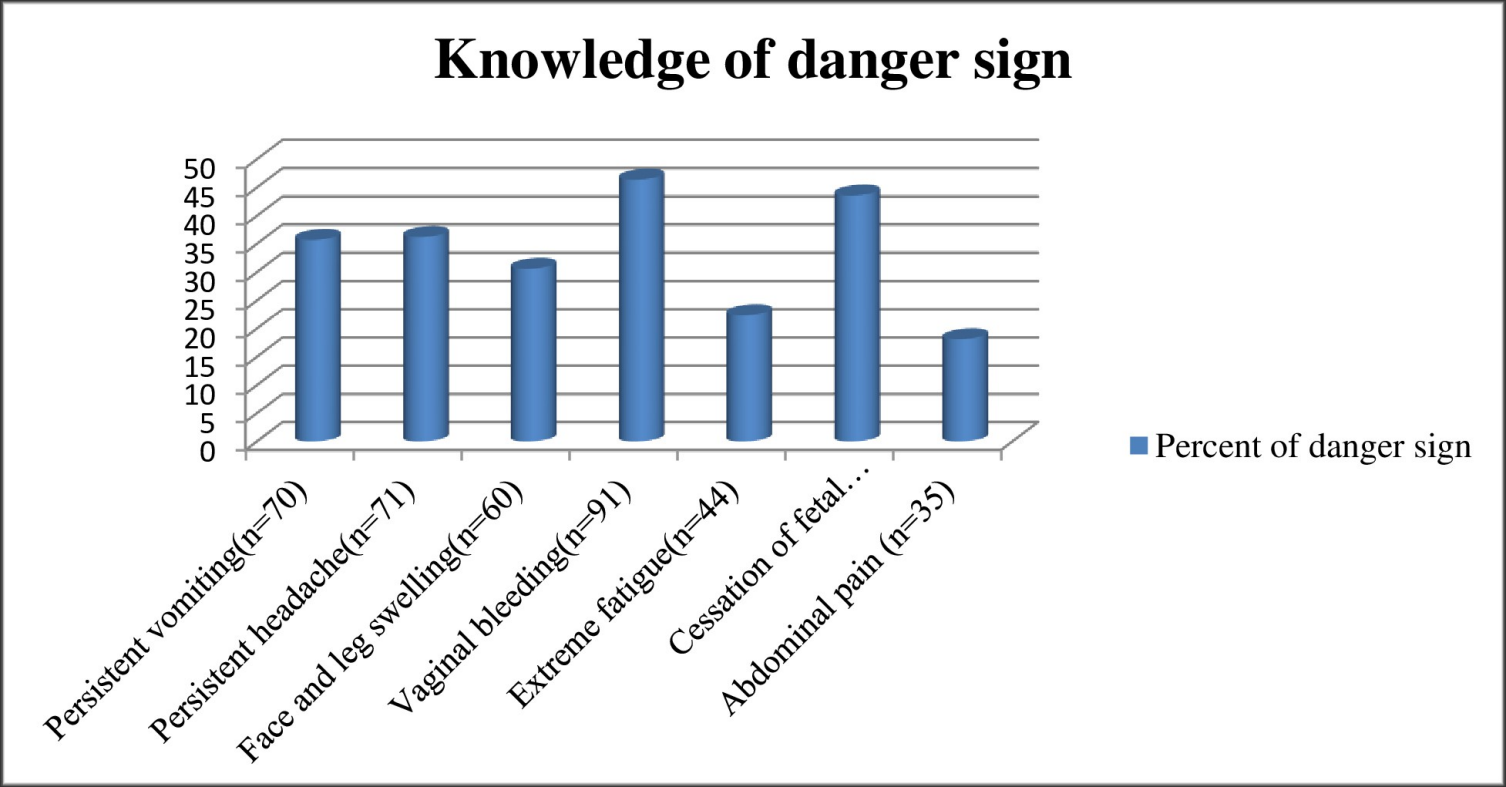
Knowledge about to danger sign in Mizan-Aman town, southwest, Ethiopia 2021.

Out of the total respondents, 395 (92.9%) thought ANC service was important for their health and 383 (90.1%) thought it was important for the fetus. Regarding the knowledge about the recommended gestational age to start ANC visits, 271 (64%) of pregnant women had an inappropriate perception of when the ANC should begin, and 154 (36.2%) had an appropriate perception of when the ANC should begin (Table 4). Of the total number of pregnant women, 196 (46.0%) of pregnant women were aware of pregnancy-related danger signs. When asked to mention danger signs during pregnancy, vaginal bleeding 91 (46.4%), cessation of fetal activity 85 (43.6%), persistent headache 71 (36.2%), and vomiting 70 (35.7%) the most commonly mentioned danger signs during pregnancy (Fig 4).

**Table 4:** Knowledge and perception of participants on the timing of ANC utilization visit health institution for ANC service in Mizan-Aman town, southwest Ethiopia 2021.

Variable	Timing of ANC initiation		Total
	Late n%	Early n%	
**Benefit ANC for a mother**			
Yes	269 (90.6)	126 (98.4)	395 (92.9)
No	28 (9.4)	2 (1.6)	30 (7.1)
**Benefit ANC for a fetus**			
Yes	257 (86.5)	126 (98.4)	383 (90.1)
No	40 (13.5)	2 (1.6)	42 (9.9)
**Perceived ANC starting time**			
Appropriate	67 (22.6)	87 (68.0)	154 (36.2)
Inappropriate	230 (77.4)	41 (32.0)	271 (63.8)
**Number of ANC visits > 4 times**			
Yes	57 (19.2)	58 (45.3)	115 (115)
No	240 (80.8)	70 (54.7)	310 (72.9)
**Awareness of danger signs during pregnancy**			
Yes	101(34.0)	95(74.2)	196(46.1))
No	196(66.0)	33(25.8)	229(53.9)

**Health service-related factors.** Out of the 425 respondents, 236 (55.5%) said that they provided ANC services during working hours in a health facility; 44.5% (189) disagreed with the full-time provision. 192 (45.2) spent four hours receiving ANC services. A majority of the 391 (92%) of them were comfortable with the ANC service they received, whereas around 34 (8%) were uncomfortable (Table 5).

**Table 5:** Health service- related factors in Mizan-Aman town, southwest Ethiopia 2021.

Variables	Frequency n (%)	Variables	Frequency (%)
**Service delivery through the working hours**		**Describe the money paid**	
Yes	236 (55.5)	Less expensive	1 (2.4)
No	189 (44.5)	Moderate expensive	34 (81.0)
**Institution distance from your home**		Expensive	7 (16.7)
Very close	135 (31.8)	**Staff approach**	
Average	210 (49.4)	Highly satisfied	38 (8.9)
Too far	80 (18.8)	Satisfied	200 (47.1)
**Transport cost**		Medium	160 (37.6)
Yes	292 (68.7)	Not satisfied	24 (5.6)
No	133 (31.3)	Highly not satisfied	3 (0.7)
**Spent maximum waiting time**		**Laboratory service**	
< 2 hours	102 (24.0)	Highly satisfied	27 (6.4)
2–3 hours	131 (30.8)	Satisfied	156 (36.7)
>4 hours	192 (45.2)	Medium	202 (47.5)
**Did you comfortable with the ANC service**		Not satisfied	28 (6.6)
Yes	391 (92.0)	Highly not satisfied	12 (2.8)
No	34 (8.0)	**Waiting time**	
**Did you return to this facility for ANC service**		Highly satisfied	12 (2.8)
Yes	339 (79.8)	Satisfied	61 (14.4)
No	58 (13.6)	Medium	164 (38.6)
I don’t know	28 (6.6)	Not satisfied	134 (31.5)
**Payment for service**		Highly not satisfied	54 (12.7)
Yes	42 (9.9)	**Privacy**	
No	383 (90.1)	Highly satisfied	15 (3.5)
**Paid for what service**		Satisfied	95 (22.4)
Laboratory	11 (26.2)	Medium	146 (34.4)
Ultrasound	27 (64.3)	Not satisfied	122 (28.7)
Drug	4 (9.5)	Highly not satisfied	47 (11.1)

#### Factors associated with late initiation of ANC

A multivariate analysis was done to identify independent predictors of the timing of the first antenatal care booking. After controlling the confounding factors, the multivariate revealed that the following factors have an association with the time of the first ANC booking: unplanned pregnancy, lack of awareness about pregnancy danger signs, inappropriate perception of ANC starting time, and pregnant women who were unaware of service delivery during working hours at the institution. Pregnant moms who did not plan for pregnancy were 2.63 times more likely than planned mothers to be late for their first ANC appointment [AOR = 2.63, 95.0% CI: 1.18, 5.85]. Women who believed that ANC bookings should begin after four months of pregnancy were four times more likely to do so than those who believed that ANC bookings should begin before four months of pregnancy (AOR = 4.1, 95% CI: 1.9, 8.83). When comparing pregnant women who didn’t recognize the pregnancy-related danger indicator to those who did, the odds of late ANC bookings were 6.76 times greater [AOR = 6.76, 95% CI: 2.83, 16.1]. Pregnant women who did not know about the service delivery during working hours in the institution were less likely to be late for ANC initiation than mothers who knew about the service delivery through time (AOR = 0.44, 95% CI: 0.19, 0.98) (Table 6).

**Table 6:** Factors associated with late initiation of ANC in Mizan Aman town, southwest, Ethiopia, 2021.

Variables		Timing of ANC initiation		COR (95%, CI)	AOR (95%, CI)	*P*. *Value*
		Late n%	Early n%			
**Residence**						
	Urban	163(54.9)	100(78.1)	1	1	
	Rural	134(45.1)	28 (21.9)	2.93 (1.82, 4.73)	2.38 (1.0, 5.68)	0.049
**Maternal educational status**						
	No formal education	93(31.3)	17(13.3)	6.12 (3.15, 11.9)	0.37 (0.069, 2.02)	0.255
	Primary (1–8)	82(27.6)	32(25.0)	2.87 (1.6, 5.13)	0.3 (0.077, 1.21)	0.092
	Secondary (9–12)	80(26.9)	32(25.0)	2.8 (1.56, 5.01)	1.05 (0.35, 3.15)	0.926
	College and above	42(14.1)	47(36.7)	1	1	
**Maternal occupational status**						
	Government employee	46 (15.5)	52(40.6)	1	1	
	Privet employee	44(14.8)	10(7.8)	4.97 (2.25, 10.99)	1.37 (0.41, 4.6)	0.602
	Housewife	82(27.6)	38(29.7)	2.43 (1.4, 4.23)	1.18 (0.359, 3.88)	0.785
	Students	55(18.5)	16(12.5)	3.88 (1.96, 7.7)	0.406 (0.093, 1.77)	0.231
	Daily laborer & Other	70(23.6)	12(9.4)	6.6 (3.17, 13.7)	1.18 (0.266, 5.27)	0.824
**Husband educational status**						
	No formal education	49 (19.2)	11 (8.7)	4.09 (1.97, 8.51)	0.469 (0.097, 2.27)	0.348
	Primary (1–8)	51(20.0)	15 (11.9)	3.12 (1.61, 6.06)	1.02 (0.277, 3.8)	0.973
	Secondary (9–12)	80 (31.4)	31 (24.6)	2.37 (1.4, 4.02)	0.89 (0.319, 2.49)	0.829
	College and above	75 (29.4)	69 (54.8)	1	1	
**Husband occupational status**						
	Government employee	71 (27.8)	71 (56.3)	1	1	
	Privet employee	24 (9.4)	7 (5.6)	3.4 (1.38, 8.46)	0.93 (0.23, 3.71)	0.922
	Private business	63 (24.7)	23 (18.3)	2.73 (1.53, 4.9)	1.23 (0.44, 3.44)	0.69
	Daily laborer	27 (10.6)	7 (5.6)	3.86 (1.58, 9.43)	1.28 (0.29, 5.88)	0.75
	Other (farmer)	70 (27.5)	18 (14.3)	3.89 (2.1, 7.18)	1.82 (0.54, 6.17)	0.33
**Planned pregnancy**						
	Yes	174(58.6)	108(84.4)	1	1	
	No	123(41.4)	20(15.6)	3.81 (2.24, 6.48)	2.63 (1.18, 5.85)	0.018*
**Receive advised to come to ANC in the current pregnancy**						
	Yes	179(60.3)	59(46.1)	1	1	
	No	118(39.7)	69(53.9)	0.56 (0.37, 0.85)	1.12 (0.56, 2.24)	0.74
**Perceived ANC starting time**						
	Appropriate	67(22.6)	87(68.0)	1	1	
	Inappropriate	230 (77.4)	41 (32.0)	7.28 (4.59, 11.54)	4.1 (1.9, 8.83)	0.00*
**Number of ANC visits > 4 times**						
	Yes	57 (19.2)	58 (45.3)	1	1	
	No	240 (80.8)	70 (54.7)	3.49 (2.22, 5.48)	1.2 (0.58, 2.45)	0.62
**Awareness of danger signs during pregnancy**						
	Yes	101(34.0)	95(74.2)	1	1	
	No	196(66.0)	33(25.8)	5.59 (3.51, 8.88)	6.76 (2.83, 16.1)	0.00*
**ANC follow- up during the preceding pregnancy**						
	Yes	125 (58.7)	73 (76.8)	1	1	
	No	88 (41.3)	22 (23.2)	2.33 (1.35, 4.04)	0.93 (0.41, 2.1)	0.87
**Service delivery through the working hours**						
	Yes	150 (50.5)	86 (67.2)	1	1	
	No	147 (49.5)	42 (32.8)	2.0 (1.3, 3.09)	0.44 (0.19, 0.98)	0.046*

*P.value<0.05, CI: Confidence interval, AOR: Adjusted odds ratio, COR: Crud odd ratio.

## Discussions

According to the World Health Organization (WHO), the first trimester of pregnancy is the recommended time for a pregnant woman to begin prenatal care. However, a significant portion of pregnant women from developing nations disregarded the advice to seek antenatal care within the first four months of pregnancy. our study found that 70% of pregnant women were late in starting antenatal care; this finding was consistent with a study conducted in African countries; in Southern Benin, approximately 75.4% of pregnant women were late in starting antenatal care [16], and in southern Nigeria, 72.4% [17]. In Ethiopia also, one multilevel analysis of EDHS showed similar findings with 67.31% of pregnant mothers delayed in attending the first ANC booking [18].

The magnitude of the late first ANC visit in this study is higher as compared to a study conducted in Cameron at 44% [19], rural women in South Africa at 51%, and peri-urban women at 28% [20]. Correspondingly, in Ethiopia Amhara region of Woldia is 59.5% [21], Debre Brihan 60% [11], and the Tigray region, is 61.4% [14]. Conducted studies found a lower magnitude. This inconsistency may be due to infrastructure, perceived ANC timing disparities, and research time differences (e.g., our study was conducted during the COVID-19 pandemic, so pregnant mothers might be hesitant to visit a health facility for ANC services and arrive late).

Our study finding is low in prevalence as compared to a study conducted in Zambia at 86.6% [22], in Tigray 85.67% [23] and in East Wollega at 81.5% [12]. This distinction may be due to participant socio-demographic features, facilities, and pregnancy classification; in our research, women who were late at or after 16 weeks of gestational age visited a health institution for their first ANC service were categorized as late bookers, which may reduce the number of late bookers.

In this study, women whose pregnancy was unplanned had a higher chance of delaying their first ANC appointment as compared to their counterparts of women with a planned pregnancy. This finding was coordinated with a study conducted in Ethiopia, Debre Markos, Ambo, and Sidama zones [23–25]. This could be due to unplanned pregnancy, which might cause shame or fear and poor readiness to go to health facilities at the recommended time.

Pregnant women may have an inappropriate perception of the ANC starting time or an appropriate perception of the ANC starting time. According to this study, people who believed that ANC reservations should be made after four months of gestation were more likely to make late reservations than people who believed that reservations should be made before that period. Likewise, another study conducted in Benin [16] and Tanzania [5] found similar findings. Mothers may consider that the ideal time to visit ANC is when the pregnancy has been physically confirmed by the family and a doctor, usually after the 16th week of GA.

The study also revealed pregnant mothers, those who didn’t have awareness about danger signs during pregnancy, had a greater likelihood of being late for their first ANC appointment compared to those aware of pregnancy danger signs. This finding was similar to a study conducted in the Sidama zone, Ethiopia [26], and Tanzania [5]. This could be due to a lack of education Most of the participants’ education status had attained primary education. This decreased awareness.

Women who know service delivery through working hours in health institutions have 2 times the likelihood of being late for the ANC, compared to those who didn’t know. This might be related to inconveniences created by the user-unfriendly, booking system, overcrowded conditions in the health care providing area, long waiting times, and health profession personnel behavior.

## Conclusion

In the current study, the majority of pregnant women (70.0%) were late in scheduling their initial ANC appointment. Pregnant women are prevented from beginning at the advised time for a variety of reasons, including an unplanned pregnancy; lack of awareness about pregnancy danger signs; inappropriate perception of ANC starting time; and pregnant women who were unaware of service delivery during working hours in the institution. Therefore, it is advised that healthcare providers provide accurate information regarding antenatal care services. It is also crucial to improve the health extension program to raise community awareness before and during pregnancy at all levels of health care provision.
